# Attitudes toward and experiences of gender issues among physician teachers: A survey study conducted at a university teaching hospital in Sweden

**DOI:** 10.1186/1472-6920-8-10

**Published:** 2008-02-26

**Authors:** Gunilla Risberg, Eva E Johansson, Göran Westman, Katarina Hamberg

**Affiliations:** 1 Department of Public Health and Clinical Medicine, Family Medicine, Umeå University, SE-90185, Sweden; 2 Centre for Gender Excellence, research programme Challenging Gender, Umeå University, Umeå, SE-90187, Sweden

## Abstract

**Background:**

Gender issues are important to address during medical education, however research about the implementation of gender in medical curricula reports that there are obstacles. The aim of this study was to explore physician teachers' attitudes to gender issues.

**Methods:**

As part of a questionnaire, physician teachers at Umeå University in Sweden were given open-ended questions about explanations for and asked to write examples why they found gender important or not. The 1 469 comments from the 243 respondents (78 women, 165 men) were analyzed by way of content analysis. The proportion of comments made by men and women in each category was compared.

**Results:**

We found three themes in our analysis: Understandings of gender, problems connected with gender and approaches to gender. Gender was associated with differences between women and men regarding behaviour and disease, as well as with inequality of life conditions. Problems connected with gender included: delicate situations involving investigations of intimate body parts or sexual attraction, different expectations on male and female physicians and students, and difficulty fully understanding the experience of people of the opposite sex. The three approaches to gender that appeared in the comments were: 1) avoidance, implying that the importance of gender in professional relationships was recognized but minimized by comparing gender with aspects, such as personality and neutrality; 2) simplification, implying that gender related problems were easy to address, or already solved; and 3) awareness, implying that the respondent was interested in gender issues or had some insights in research about gender. Only a few individuals described gender as an area of competence and knowledge. There were comments from men and women in all categories, but there were differences in the relative weight for some categories. For example, recognizing gender inequities was more pronounced in the comments from women and avoidance more common in comments from men.

**Conclusion:**

The surveyed physician teachers gave many examples of gender-related problems in medical work and education, but comments describing gender as an area of competence and knowledge were few. Approaches to gender characterized by avoidance and simplification suggest that faculty development programs on gender need to address and reflect on attitudes as well as knowledge.

## Background

During the last decades, research has shown unexplained differences in the treatment of women and men in various areas of clinical and academic medicine. These reports have led to a growing awareness of gender bias in medicine and a discussion of how to avoid it [1-11]. One way to minimize such bias would be to introduce gender issues and apply a gender perspective in medical education [11,12]. Physician teachers are in a position to influence the success of this kind of work.

The concept of gender has been used in the social sciences and humanities since the 1960s. It was originally introduced to describe how different societies and cultures interpret biological sex [13]. Currently the term gender refers to the constantly ongoing social construction of what is considered 'male' and 'female' based on sociocultural norms and power. The power asymmetry between women and men has been conceptualized as the 'gender order' [14], a structuring principle in society characterized by separation and hierarchy. Gender indicates that differences between women and men are not to be seen as naturally occurring or unchangeable. Instead gender is subject to change and negotiation. We all 'do gender' in all kinds of social interactions [15]. A gender perspective in medicine would imply that life conditions, positions in society, and societal notions about 'femininity' and 'masculinity' should be considered along with biology in all professional interactions, as well as when theorizing about women and men [10,16].

We wanted to investigate potential prerequisites and/or problems when we were engaged in implementing a gender perspective into the curricula of the Umeå Medical School in Sweden in the late nineties. Therefore, we surveyed physician teachers' attitudes toward the importance of gender in various professional relationships, such as in consultation and in clinical tutoring. In the statistical analysis of ratings obtained in this study, we found that the gender of the physician influenced these attitudes. Female physician teachers found gender important to a significantly higher degree than did the men [17]. As a part of this survey, open-ended questions were asked about attitudes. This paper analyzes the answers given to these questions.

Our objective was to gain a better and more nuanced understanding of physicians' attitudes toward gender issues than we got from the ratings mentioned above. To find suitable ways to engage both women and men in the implementation of gender, we also aimed at comparing comments from female and male physicians.

## Method

### Design

We sent a questionnaire to all 468 specialists, 29% of whom were women, who were employed in the clinical departments of the university hospital or in family medicine in Umeå, Sweden. All were engaged in teaching medical students and/or tutoring them in their clinical training. The procedure and participants have been described in more detail in earlier papers [17].

The physicians were asked to rate their degree of agreement with five statements about the importance of gender in different professional relationships (Table 1) on a 100 mm visual analogue scale. Below each statement there were two open-ended questions, one asking for explanations for why they found gender relevant or not, and the other for examples and situations where gender might be of importance. There was a space of five centimetres for each answer.

**Table 1:** Statements on the importance of gender

1. The patient's gender is of importance in consultation.
2. My own gender is of importance in consultation.
3. The gender of the medical student is of importance in clinical tutoring.
4. My own gender is of importance in clinical tutoring.
5. My own gender is of importance in my professional relationships, for example with colleagues, medical staff or in research.

The Umeå Clinical Research Ethics Committee approved the study.

### Analysis

The answers were given numbers and transcribed verbatim into computer text. When reading and coding the transcripts it was not possible to see whether the respondent was a man or a woman, but the numbers made it possible to later sort comments according to whether they were written by a man or a woman.

The textual elements were analyzed using content analysis [18,19]. In the first step, the comments were read through by all four researchers, and coded openly according to content and meaning. The sets of codes were then compared and discussed and preliminary categories were outlined. These were refined in a second step when all the comments were reread and coded by the first author. By constant comparisons and discussions the codes were condensed into eight main categories. These in turn were grouped into three themes.

In the next step all 1 469 comments were sorted in the categories found. Some comments illustrated more than one category and they were sorted accordingly in two, or in a few cases, three categories. Difficulties in sorting particular comments were discussed by all the researchers. Answers that could not be categorized were marked as miscellaneous.

## Results

The questionnaire had a response rate of 65% (n = 303: 92 women and 211 men). Of these respondents 85% (n = 78) of the women and 78% (n = 165) of the men gave answers to one or more of the open-ended questions. In total, there were 565 comments from women and 904 from men. Thus the data consisted of 1 469 short textual elements, varying from a couple of words to several handwritten lines.

Three main themes were identified in the physicians' comments: *understandings of 'gender'*, *problems connected with gender*, and *approaches to gender*. An example of how comments were coded and condensed and labelled in categories, and of one emerging theme is presented in Table 2.

**Table 2:** Comments and the emerging codes, categories and themes in the content analysis.

**Comments**	**Codes**	**Categories**	**Themes**
*"Women and men can never really understand each other." (male, 47, general practitioner)* *"This is rather obvious considering that women and men express themselves in altogether different manners." (male, 47, non-surgical specialty)*	Men and women behave differently	Differences	**Understandings of 'gender'**
*"Coronary disease in women differs from coronary disease in men with regard to symptoms, seriousness and treatment risks."(female, 38, non- surgical specialty)* *"Men and women describe symptoms differently and expect different therapies" (male,42, general practitioner)*	Women's and men's diseases are different
*"It is an advantage to be a man. You have more authority in contact with staff and with patients."(male, 41, surgical specialty)* *"Maybe I listen more to the demands about investigations and treatment from male patients and see women's symptoms as less important, easier to ignore?" (female, 41, general practitioner)*	Hierarchies and injustice	Inequity
*"It is probably harder to get a picture of the total life situation of the female patient than the male"(female, 46, non- surgical specialty)* *"A women's life is partly something else and this affects health and should affect interpretation of symptoms." (male, 51, surgical specialty)*	Men and women have unequal life conditions and experiences

After the coding and categorization of the anonymous comments were finished, we broke the number code and identified whether an answer was given by a man or a woman. We thereafter sorted and counted the number of comments from women and men in each category. The relative proportion of comments from women and men respectively in each category is presented in the first section of Table 3. In the second section of Table 3 the number and percentage of respondents who had comments in each category are presented, women and men respectively.

**Table 3:** Themes, categories and codes from the content analysis

	Number of comments in the categories as well as the relative proportions of comments in each category, women respective men (column in %)			Number (and percentage) of respondents who had comments in each category, women respective men		
**Themes**, *categories *and codes	Total N = 1 469	From women N = 565	From men N = 904	Total N = 243	Women N = 78	Men N = 165
**Understandings of 'gender'**	n (%)	n (%)	n (%)	n (%)	n (%)	n (%)
*Differences*	394 (27)	156 (28)	238 (26)	162 (67)	53 (68)	109 (66)
Behaviour						
Disease						
*Inequity*	291 (20)	175 (31)	116 (13)	115 (47)	57 (73)	62 (38)
Life conditions and experiences						
Hierarchy and injustice						
**Problems connected with gender**						
*Delicate situations*	123 (8)	33 (6)	90 (10)	75 (31)	26 (33)	49 (30)
Embarrassing situations						
Sexual attraction						
*Gendered expectations*	53 (4)	35 (6)	18 (2)	48 (20)	30 (38)	18 (11)
Male norms						
Women are caring						
*Lack of experiences*	17 (1)	7 (1)	10 (1)	16 (7)	6 (8)	10 (6)
**Approaches to gender**						
*Avoidance*	288 (20)	70 (12)	218 (24)	118 (49)	35 (45)	83 (50)
Minimize importance						
Doctors are neutral						
Not me – others						
*Simplification*	151 (10)	50 (9)	101 (11)	83 (34)	30 (38)	53 (32)
Self-evident						
A question of teaching women						
Equity already achieved						
*Awareness*	124 (8)	63 (11)	61 (7)	74 (30)	34 (44)	40 (24)
Social conditions make difference						
Intersectional factors important						
Lack of female role models						
Gender as competence						
**Miscellaneous**	77 (5)	24 (4)	53 (6)	57 (23)	14 (18)	43 (26)
	1518*	613*	905*			

* Some comments concerned more than one category and thus the summary of the columns 1, 3 and 5 exceed N in table head.

The three main themes and the categories underlying them are presented below and exemplified with comments. No physician is quoted more than once. Information about gender, age and specialty group of the respondent is provided in brackets following each comment. The specific specialty is not presented for anonymity reasons. (The surgical specialty group includes anesthesiology and intensive care, general surgery, pediatric surgery, hand surgery, neurosurgery, orthopedics, plastic and reconstructive surgery, thoracic surgery, urology, obstetrics and gynecology, gynecological oncology, ophthalmology and otorhinolaryngology. The non-surgical specialty group includes pediatrics, dermatology – venereology, general internal medicine, endocrinology, infectious diseases, respiratory medicine, nephrology, rheumatology, geriatrics, occupational & environmental medicine, clinical physiology, transfusion medicine, clinical neurophysiology, neurology, psychiatry, child & adolescent psychiatry, community and social medicine, diagnostic radiology, oncology, rehabilitation medicine and clinical genetics.) Ethnicity is not provided because in this respect the study group was homogenous; all but a few were white and Swedish.

### Understandings of 'gender'

There were two categories within this theme: Difference and inequity.

#### Difference

Many examples in the open-ended comments were about differences between women and men, regarding *behaviour *and *diseases*.

Female and male doctors, patients and students were described as thinking and acting in different ways. One example was the physician teachers' comments on students. When describing female students, recurring characteristics were: low self-esteem, cautious, listening, empathetic, and capable. Male students were described as rational, confident, demanding, provoking, pushy, competitive, as overestimating themselves and as taking up a great deal of space.

"Female students often underestimate themselves. This indicates that when tutoring you have to instruct female students 'how to fly' and male students 'how to land'." (female, 41, unknown specialty)

Differences between male and female patients and male and female doctors were also described, as exemplified in this statement:

"It is easier to talk to male colleagues because we share views on how to investigate and treat patients in most cases. There are fewer irrational factors than in contact with female colleagues." (male, 59, non-surgical specialty)

Attitudes, values, ways of thinking, ethics, language, and symptom presentation were some of the behavioural fields that were repeatedly described as being different between women and men.

Statements on differences concerning diseases consisted of remarks on different health problems, symptoms, and epidemiology. Several diseases and symptoms were mentioned, such as rheumatism, thyroid diseases, psychiatric symptoms and chronic pain. The teachers also commented on the fact that female and male patients are investigated and treated in different ways. Comments about cardiovascular disease were particularly frequent.

"Patterns are different for men and women with coronary heart disease. What causes stress for men is not the same thing that causes stress for women and vice versa." (male, 44, general practitioner)

A few teachers commented on sameness between women and men. When they did, they paired similarities with differences.

"There are both similarities and differences between men and women. Both phenomena must be thought about in medical education." (male, 49, general practitioner)

#### Inequity

We found the perception of inequity expressed as numerous descriptions of existing power asymmetry, discrimination, *hierarchies and injustice*, where women were most often identified as disadvantaged.

"It is a shame that women who are more sensitive and suffer more from pain are often paid no attention, and are even humiliated during consultations for pain." (female, 43, non-surgical specialty)

There were also comments about power structures that are hidden or closed for women.

"Men often have meetings outside work, meetings to which female researchers/physicians are not invited." (female, 43, general practitioner)

There were remarks about how men are looked upon as having a higher value than women and get more respect.

"When new students, both male and female, turn up at a ward they often shake hands politely with the males in the room – quite unconsciously. There might be four female doctors and one male nurse present." (female, 59, non-surgical specialty)

There were also examples related to inequality of *life conditions *and *experiences *for women and men, such as different domains of paid work. Sexualized violence, as part of the life experiences of many women, was also considered. Most comments were about women's unpaid work and their greater responsibility for children, family and domestic duties.

"During the last decades, it has become increasingly important to ask female patients about the situation both at work and at home. Many women are very strained by their double commitments." (male, 63, surgical specialty)

Women understood 'gender' as a question of inequity to a higher degree than men. Thirty-one percent of women's comments concerned inequity compared to 13% of the men's, and 73 % of the women respondents commented on inequity while 38% of the men (see Table 3).

### Problems connected with gender

The answers dealing with problems connected with gender were about delicate situations, gendered expectations and lack of experiences.

#### Delicate situations

Several comments dealt with *embarrassing *situations associated with the body and body examination. Gynaecological examinations were often used as examples as were examinations of male reproductive organs.

"Rectoscopy can be awkward for a man when the doctor is a woman." (female, 42, surgical specialty)

Encounters with patients who have been victims of sexual abuse were also recognized as delicate situations where respect for the integrity of the patient was identified as most important.

Our data also revealed some problem descriptions, mainly from men, connected with *sexual attraction *and objectification. Examples given were mainly between doctor-patient or teacher-student. The physicians commented on being the object of admiration and attraction from patients or students of the opposite sex, but there were also stories about themselves being attracted.

"You get help from me only because you're attractive." (female, 50, general practitioner quoting a male colleague)

"You are only human. A beautiful and elegant woman will of course be treated more cordially by a male doctor, at least in a stressful situation." (male, 50, non-surgical specialty)

#### Gendered expectations

Women especially commented about specific expectations for female doctors, since *male norms *surround physicianship. The women's comments dealt with repeatedly having to prove that they were as capable and skilled as male doctors.

They also described that women were expected *to be caring *and empathetic and to participate in the social activities of the workplace.

"As a female doctor I am expected to take a large responsibility for patients and to show great respect to the staff. For example, cleaning the examination room, having the strength to listen and to be caring, and having "difficult" patients as well as extra patients." (female, 56, non-surgical specialty)

Some male doctors also commented on the expectations of the female doctor.

"I think I would be questioned more in my work by the patients if I were a woman. They never take *me *for a nurse." (male, 44, surgical specialty)

A few men mentioned specific expectations of them being male doctors, such as being a trouble-shooter.

A higher proportion of female teachers (38%) than male (11%) commented about gendered expectations (Table 3).

#### Lack of experiences

A few comments dealt with the problem and difficulty in imagining what it is like to be in the body of the opposite sex. In this small category, we also sorted comments that concerned lack of gender-specific experiences, such as men not being able to experience symptoms related to pregnancy.

### Approaches to gender

This theme consisted of three different ways to tackle the subject of gender, i.e., approaches of avoidance, simplification and of awareness.

#### Avoidance

Many respondents, men to a greater degree than women, *minimized *the importance of gender (figures not shown in table 3) by declaring that something else – not gender – was important, e.g., interest, knowledge and empathy of the physician, or personality of the patient and of the physician (Table 3). A handful of the minimizing comments from men also disclosed irritation, for example:

"Gender is a trivial subject engaging only dissatisfied upper-class women". (male, 39, surgical specialty)

Other comments that were categorized as avoidant described the doctor's role per se as objective and *neutral*, and gender issues were therefore less important:

"I am solely a professional – neutral and genderless." (male, 40, surgical specialty)

Other ways to diminish the importance of gender was to comment that *I myself *have *no problems *with gender in professional relationships, or by emphasizing that in my specialty we are gender neutral.

"My professional interest is dementia. Therefore I find the question of gender irrelevant." (female, 59, non-surgical specialty)

The importance of gender was also reduced in statements making it a matter mainly of *other *cultures:

"Gender might be important for some foreign patients. Male Arabs for example don't want to be examined by female professionals. Female Muslims sometimes do not accept male staff." (male, 61, non-surgical specialty)

#### Simplification

Several short comments expressed the view that the importance of gender issues is so *self-evident *that there is no need to focus on these issues in medical education and professional relationships.

Another way to simplify was to argue that gender is all a matter of *teaching women *certain things, for example how men think and speak, to say no, to take space – but still do it 'as a woman'.

"Teach women that the goal is not to become like a man but a wonderful, capable woman!" (male, 48, surgical specialty)

No examples were given about teaching men.

Some physicians argued very strongly that *equity is already achieved *between men and women in our society. Consequently, it was claimed, men and women are (or should be) treated equally. These arguments often maintained that individuals should be looked upon as human beings, not as a man or as a woman.

"I would like to think that we are all equal human beings and can understand each other." (male, 50, non-surgical specialty)

#### Awareness

Comments reflecting on *psychosocial conditions *and *sociocultural norms*, relation and power were categorized as reflecting an awareness of gender.

A few physicians considered how professional relationships in the medical world mirror society in general. Some women commented on how their own gendered patterns contribute to the upholding of unfair differences and discrimination.

"I do not demand assistance in everyday tasks; nor do I ask for help from nurses and secretaries as my male colleagues do." (female, 47, general practitioner)

Another way of thinking was to discuss how other hierarchical categories like social class, ethnicity, position and age *intersect *with gender to mediate possibilities and choices for individual men and women.

"In the same way as age, language, nationality and social class, gender is of importance in the consultation. In all clinical situations these factors can lead to discrimination if the physician lacks self-knowledge and is unacquainted with empathy and does not demonstrate a professional attitude." (male, 43, surgical specialty)

Several women and one man reflected on the lack of women *role models *for female students. The women physicians had no female role models during their own education.

"Role models are important. I had no female role models myself." (female, 44, general practitioner)

No one mentioned the need for male role models.

There were sporadic comments on *gender *issues as an area of *competence *and knowledge, expressed as a wish to know more about gender theory and gender research.

"There is not enough understanding of problems emanating from deeply rooted expectations and norms about men and women."(male, 56, non- surgical specialty)

"Gender is a low status area. I need to learn more. I have missed deeper insights." (female, 39, general practitioner)

Only eight percent of the comments reflected awareness but such comments were given by 30 percent of the teachers (44 percent of the women and 24 percent of the men.)

## Discussion

### On findings

This presentation analysed how physician teachers consider, reflect on, and explain their attitudes toward gender issues. They understood gender as a question of differences and inequity between women and men. They gave examples of problematic situations in working relationships that were related to gender, such as embarrassment, attraction and difficulties in understanding the experience of people of the opposite sex. Their comments also demonstrated three different approaches to gender issues; avoidance, simplification and awareness. Women commented to a higher degree than men about inequity and gendered expectations and men to a higher degree than women demonstrated avoidant approaches.

#### Differences – sex-related characteristics or a question of inequity?

In comments from two thirds of the physicians, gender was associated with difference. On one hand, these dealt with differences in diseases, which is not surprising considering the medical context. On the other hand, many comments also referred to rather dualistic differences in the behaviour of women and men. This mirrors societal attitudes in general.

Dichotomous views of men and women have a long history in our culture. Such gender stereotypes can lead to gender bias and discrimination. Men and "manliness" have been looked upon as the norm for mankind while women and "womanliness" have been perceived as aberrant. These traditional ideas have been reflected in medicine, as illustrated in a classical investigation from 1970; American psychiatrists and psychologists were asked to describe a healthy man, a healthy woman and a healthy adult respectively. From a list of alternative adjectives they chose similar words to describe a healthy adult and a healthy man, for example active, logical and autonomous. To describe a healthy woman opposite words, like passive, illogical and dependent were more often used [20]. Similar results have been reported from the late 1990s. At a Canadian medical school, students equated adults with men and saw women as "others" [21]. In Sweden course organizers at a medical school used contrasting characteristics when they described female and male students [22] as did supervisors in physiotherapy when describing physiotherapy students [23].

In line with this, many of the comments in our study, where men and women were described as having different values and behaviour, came rather close to using gender stereotypes. This language of dualisms might have been exaggerated by the fact that the teachers were asked to explain their viewpoint. The differences between women and men were often formulated as innate and as something to appreciate, not to alter. This might imply that men and women are seen as separate, characterized by complementary relationships. This view can lead to an inability to deal with power and inequity, and thereby counteracts work toward gender equity, which still is a critical issue in academic medicine [6,7].

Research has shown that power differentials between women and men are often disregarded in the medical context. In a Swedish interview study of doctors, nurses and auxiliary nurses on professional identities, notions about femininity and masculinity were expressed without seeing the existing gender order as problematic [24]. In another interview study with teachers and students at a medical school, the researchers found that knowledge of the gender system and its power structures was not sufficiently taken into consideration in the patient-doctor relationships or in teaching about health and sickness [25].

Our informants, however, – women to a much larger degree than men (see Table 3) – also perceived gender as a question of inequity regarding position and power. In those comments, there were reflections on the risk of injustice if different positions and gendered norms are not taken into consideration, as shown for example by the tendency to investigate male patients more thoroughly than female patients. Wishes for change were expressed. We see this as an awareness of a gender order [14] and how it permeates into behaviour and relationships, professional as well as private.

#### Resistance against gender issues?

Another important finding in this study was that 30 percent of the comments expressed avoidant and simplistic standpoints on the subject of gender, which might imply a lack of awareness of and interest in gender issues. Nearly half of the teachers provided such comments.

One example is that the physicians used neutrality as a critical argument for modulating the importance of gender in professional relationships. This argument might originate from the construction of the traditional physicianship, where decisiveness, authority, rationality and objectivity are important characteristics [25-27]. Objectivity includes perceiving oneself as gender neutral; thus gender could be disregarded in professional relationships. A survey and interview study among students and faculty in a Canadian medical school indicated that gender neutrality was still an important component in the professional socialization of future physicians [28].

There were also many comments that men and women are (or at least should be) treated alike nowadays in our society, indicating that there is no need for gender issues to be on the agenda. Such arguments can be understood as ideological proclamations. They are politically correct and fit comfortably in the Swedish political discourse saying that women and men are equal and are treated equally. Such a discourse denies the existence of a gender order.

Only a few comments in our open-ended answers dealt with gender as a competence area. We interpret this to mean that many physician teachers are unaware of and might need information on gender as a scientific field [29]. This interpretation is supported by research reporting that medical students sometimes find education about gender of low theoretical level and that there is a risk that gender stereotypes are recreated in the discussions [12]. Prolonged and sometimes difficult work has been reported from teachers trying to implement a gender perspective into medical curricula [12,30-32]. The different manifestations of resistance to gender issues that we found among physician teachers might be one explanation.

#### Gender – an issue for women only?

Both men and women made comments that fit into all the themes and categories of our analysis. Women, however, commented much more than men on inequity, for example on differences in experiences and different life conditions. This probably reflects women's greater personal experiences of, and reflections on these subjects than men's, as illustrated for example by their explicit advice about the need for female role-models. Also, a higher proportion of women (44%) than men (24%) had comments reflecting awareness.

More of the avoidant and simplistic comments came from men, and only men gave comments that indicated irritation. This is in line with the results on the scaled responses; men found gender less important than women [17].

Understanding gender issues as women's issues could be understood from a hierarchical perspective. Women's subordination in the gender system can explain why the specific problems and difficulties women meet have dominated gender research and gender studies for a long time. Consequently, in medicine the situation of female patients and female professionals has been the focus. However, gender issues and gender relationships involve men as well as women. Masculinity norms, just like femininity norms, imply restrictions and entail specific consequences, challenges and difficulties. For example, it has been discussed how gendered expectations of men can lead to risk behaviour and risk exposures resulting in higher mortality and poorer quality of life compared to women [16,33,34].

We argue that if medical students are to recognize the importance of learning about gender, then this should also be an issue for men. They need knowledgeable and interested men as well as women as teachers on the topic. For example, different researchers have shown that a strong, clear commitment from faculty leaders and heads of departments created a climate where faculty members of both sexes felt engaged and encouraged to learn more about gender [35,36].

### On method

The analysis of open-ended comments in a questionnaire on attitudes to gender has certain limitations. Firstly, the results are dependent on the respondents' willingness to provide examples and explanations. Secondly, the answers might be distorted due to the actual assessment of the degree to which they found gender important in professional relationships. Although the questions were open-ended, they were linked to particular statements which might have suggested certain answers. We have therefore considered the risk of emphasizing decontextualized comments in our analysis by interpreting the comments as expressions of a general gender discourse rather than linking them to specific physicians. Another shortcoming is that our design did not enable us to follow up viewpoints or get clarifications of very short comments. Interviews with physician teachers would have allowed that and perhaps made interpretations more credible [37]. Therefore, we plan to do further research on this topic by way of in-depth interviews.

Still, the large number of respondents and comments has provided us with rich data. Coding the many text elements provided categories with variation and nuances, and it also gave negative examples. Hence, we were able to identify a great range of categories that helped us to better understand attitudes toward gender issues, which was our intention.

By counting how many comments that could be sorted in each category, and determining whether comments were from male or female physicians, we wanted to give a rough idea about the relative weight of each category. The figures are not meant to be the basis for statistical tests or generalisations to a population. The aim with this content analysis was to provide new understanding of certain phenomena, and raise fruitful hypotheses. Still, the results are context bound. The phenomenon explored in this paper was physician teachers' attitudes toward gender issues. These attitudes came from physician teachers at a medical school in Sweden – a country which shares much of cultural history, norms and traditions with the rest of the western world. Thus, we believe that the findings might provide ideas that can be used elsewhere when trying to implement gender into medical education and curricula.

## Conclusion

This study showed that the physician teachers understood gender as a question of differences and inequity between women and men. The respondents gave many examples of gender-related problems in medical work and education; however few comments described gender as an area of scientific knowledge and competence. Although there were many insightful examples presented, the teachers' reasoning about the importance of gender issues in their own work revealed approaches of avoidance and simplification. There were many similarities between men's and women's comments but more women wrote about inequity than men, and avoidance was more frequent in men's comments. Avoiding and simplifying might signify a lack of awareness of gender issues, or might reflect a quiet resistance to gender, which can explain the difficulties and obstacles reported when trying to implement gender issues in medical education. This study suggests that faculty development programs on gender need to support both male and female teachers' participation and address attitudes along with knowledge about gender. In our own gender courses and development programs for teachers we have used group discussions and role-plays about tutoring as practicable ways to address gender attitudes. Engaging the participants in writing short papers about concrete gender aspects of their own teaching areas and acting as opponents of each other's papers, has been another successful way to approach and discuss attitudes.

## Competing interests

The author(s) declare that they have no competing interests.

## Authors' contributions

All four authors contributed to the conception, design, and drafting of this article. All authors have read and approved the final manuscript.

## Pre-publication history

The pre-publication history for this paper can be accessed here:


